# Evaluation of the Effectiveness of Protective Patches on Acupoints to Preserve the Bioenergetic Status against Magnetic Fields

**DOI:** 10.1155/2018/4732130

**Published:** 2018-09-17

**Authors:** Claudio Molinari, Ian Stoppa, Nicola Limardo, Francesca Uberti

**Affiliations:** ^1^Laboratory of Physiology, Department of Translational Medicine, University of Piemonte Orientale, Via Solaroli 17, Novara 28100, Italy; ^2^Health Sciences and Oral Hygiene, The Lifestyle Medicine, University “La Sapienza”, Piazzale Aldo Moro 5, Roma 00185, Italy

## Abstract

The potentially harmful nature of electromagnetic fields (EMF) and static magnetic fields (SMF) has become a major problem in recent years. All these elements could be combined to produce cellular responses. For example, the orientation of molecules of water or other complex molecules, growth and cell viability, cell morphology, and intracellular metabolic pathways have demonstrated binding to magnetic fields. The effect of EMF and SMF on humans is a topic of great importance, especially because modern technology has introduced artificial magnetic fields such as those generated by power lines, mobile communications, and medical imaging equipment. A relevant problem is certainly that of professional exposure. The aim of this study was the evaluation of the effectiveness of a commercially available device, Skudo® patches (Edil Natura S.r.l., Novara, Italy), in protecting magnetic resonance operators from the influence of magnetic fields such as those present in the workplace. Skudo® patches are designed to protect microareas of the body from external electromagnetic disturbances. In this study, 10 male Italian volunteers aged between 50 and 60 were enrolled in the hospital. All participants were subjected to measurements at 4 specific time points to evaluate the effectiveness of Skudo® to counteract both EMF and SMF magnetic fields by evaluating the level of bioenergetic reactivity. To perform the measurements, a variant of the Ryodoraku method has been used, based upon the assessment of electropermeability. In particular, 12 acupoints were measured, one for each of the main meridians. This study shows that both SMF and EMF cause an alteration of the body's water system. The application of Skudo® patches determines a regularization of bioenergetic levels related to the water system. The application of Skudo® on the EMF source has suppressed the imbalance effect of the water system found in the subject without any protection.

## 1. Introduction

A growing body of evidence has shown that magnetic fields have the ability to interact with biological systems and to induce effects in the living matter. This topic has long been of interest in the scientific community both for its applicability in the therapeutic field and for determining whether they could be potentially harmful.

The potentially harmful nature of magnetic fields has become a serious problem in recent years due to the enormous increase in the number of electronic communication devices and also the increasing use of NMR in medicine [1]. During the evolution of life on Earth, living organisms have constantly been exposed to the geomagnetic field. On our planet it can vary from 20 to 70 *μ*T. For this reason, the biological systems have developed specific mechanisms for the perception of the natural electric and magnetic fields involved, for example, in the orientation and migration of some animal species [2]. The mechanisms of detection and response to both electromagnetic fields (EMF) or static magnetic fields (SMF) can be found at different levels, for example, on the cell membrane or within a tissue. Sometimes the sensitivity of a biological system to SMF is expressed through changes in the signal transduction cascade or nerve tissue activity [3, 4]. A recently studied aspect is the effect of low-intensity SMF on cell production of free radicals. Both reactive oxygen species (ROS) and nitrogen (RNS) were studied. ROS and RNS play significant roles in immunological defense [5], intracellular signaling [6], and intercellular communication [6]. It is assumed that EMF and SMF could change the duration of radical pairs. A radical pair consists of two radicals that have been created simultaneously, usually by a chemical reaction, and possesses magnetic properties. If an SMF affects cells through the radical pair mechanism, it can influence the rotation of electrons in free radicals, which can lead to changes in the kinetics of the chemical reaction and possible alterations of cellular function [7]. Most of the studies on the biological effects of SMF considered only low-intensity stimulations. Unfortunately, the effects of strong SMFs have not yet been evaluated sufficiently, although it is easy to think that even a strong SMF should have the ability to influence biological systems. Actually, the results of these studies are controversial. Sirmatel et al. [8] studied the effects of a high-intensity magnetic field produced by a magnetic resonance imaging (MRI) apparatus on oxidative stress. However, in this study, SMF does not seem to produce a negative effect; on the contrary, it has produced the positive effect of a decrease in oxidative stress in men after short-term exposure. On the other hand, a Nakagawa study in mice showed that [9] high-intensity SMF exposure induces increased peroxidation levels in the liver of mice and also enhances the effect of hepatotoxic substances such as carbon tetrachloride, CCl_4_.

The cell is certainly a complex system formed by a set of components susceptible to the presence of EMF and SMF, such as electrical charges and molecules with their magnetic moment [10]. All these elements could be combined to produce cellular responses and, since the cellular environment includes a nonlinear system, magnetism-dependent phenomena could result from the combination of many conditions. For this reason, especially in the last decade, the behavior of cellular structures and characteristics has been studied following exposure to SMF. For example, cell and intracellular component's orientation, cell growth and viability, cell morphology, enzymatic activity, and biomolecules synthesis were investigated during exposure to SMF. For a review, see [10]. Based on numerous studies, a link between magnetic fields and observed cellular responses can be hypothesized. The mechanisms underlying these effects could be magnetosome presence, spin modulation of radicals, drifting of molecules in buoyancy following a magnetic field gradient, torques in molecules, linkage of ion to the enzyme active site, ion-protein attachment [11], calcium mobilization and diamagnetic anisotropy of lipids, mitochondria, DNA helix, and cytoskeleton.

Unlike SMF, EMF are waves that transport energy through space. Wavelength and frequency are the main features of EMF, and they are inversely correlated. Electromagnetic radiation is distributed in a spectrum ranging from radio waves to gamma rays, passing through visible light and microwaves. Starting from ultraviolet EMF are ionizing and produce damage to living organisms. EMF with frequency lower than the ultraviolet can induce thermal and nonthermal modifications in biological systems [12, 13].

The effect of EMF and SMF on humans is a topic of great importance, especially since the modern technology has introduced artificial magnetic fields such as those generated by power lines, mobile communications, and imaging equipment. In fact, the exposure to radio frequency and microwave electromagnetic fields, both in the work and in the general environment, has never experienced a growth like the one seen in the last 10 years. For this reason, it is of fundamental importance to address the problem of safety, using all the tools available to evaluate the potential risks of exposure. Moreover, a relevant problem is certainly that of occupational exposure. For example, individuals working in proximity of an MRI device are exposed to SMF coming from the scanner magnet. An important group of people regularly exposed to electromagnetic fields related to magnetic resonance imaging is the healthcare staff. Some publications describe subjective symptoms related to exposure to SMF, reported by people who have been exposed to MRI-related fields such as health personnel, patients, or healthy volunteers [14–17].

The evidence comes from both experimental and observational studies and includes general symptoms such as headaches and concentration problems, as well as specific sensory symptoms such as vertigo, balance problems, nausea, metallic taste, and flashes of light. Current literature suggests that these symptoms have an acute and transitory nature [17, 18], and many of these occur when people move through spatial gradients in the external static magnetic field outside the MRI scanner [19].

Although there is no clear evidence of a direct relationship between EMF or SMF and disease, some efforts have been made to try to develop protective methods or devices. Nowadays there are commercially available devices that claim to be able to screen the potential harmful effects of both static and electromagnetic magnetic fields. However, studies that evaluate the effectiveness of these devices in protecting the human body are still lacking. The purpose of this study was therefore the assessment of the effectiveness of one of these commercial devices in protecting MRI operators from the influence of magnetic fields present in the workplace.

## 2. Materials and Methods

### 2.1. Protective Device Tested

In order to carry on this study, we decided to test the effectiveness of a new and hi-tech device, in the form of patches, called Skudo® (Edil Natura S.r.l., Novara, Italy). The Skudo® patches are designed for the protection of microareas of the body from external electromagnetic disturbances, and each beneficial effect observed should be considered “indirect”. They are composed with a base in Pe-eVA (transparent polyethylene foam) and with nonstick gauze. In addition, they have circular shape with a diameter of 25 mm and optimal weight and thickness (60 g / m2 of weight and 70 thick micrometers). The production process has been patented and certified at European level (European Patent Certificate No. 2073611). The effectiveness of each patch is about 12 hours and it can be used by everyone regardless of age or gender. These patches are placed on the “energy points” of the meridian channels of body. If these points are effectively protected by environmental perturbations such as artificial electromagnetic fields and natural radioactivity, they can provide many beneficial effects to the body. The Skudo® patches protect these meridians by forming a physical barrier against environmental factors. These patches do not release substance, are not transdermal, have no side effect, and have no time limitations.

### 2.2. Subjects

In this study, 10 male Italian volunteers were enrolled within the hospital between 50 and 60 years of age, subject to written authorization at the Physiology Laboratory of the University of Piemonte Orientale (Novara, Italy). The approval for this study was conferred by the local Human Investigation Committee. To ensure the homogeneity of the study, the following exclusion criteria were applied: body mass index (BMI) <18.5 kg / m2 or> 30 kg / m2 [20]; autoimmune diseases; skin allergies; hypertension; surgery or critical medical history within the year prior to the study; metallic implants in the body; chronic diseases; contraindications for electrical stimulation; the inability to complete a form; and any other factors that the investigator judged to be inappropriate for the study. Then they were randomly assigned to either the control group or the Skudo® group. All participants did not receive any training or equipment to use.

### 2.3. Assessment of Body Energetic Status

**Figure 1:**
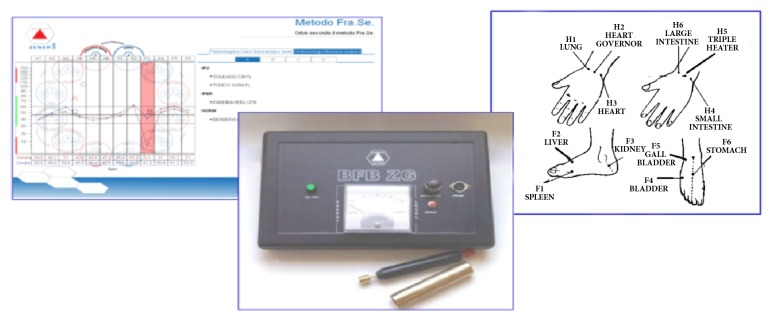
Experimental equipment. A schematic BFB-Z application and interpretation.

The state of health corresponds not only to the biochemical balance but also to the electric equilibrium. In this study, the level of health was observed by evaluating the electropermeability of some points taken from the acupuncture meridians. The equipment used was the BFB-Zener (Zener S.r.l., Milan, Italy; BFB-Z), as reported in Figure 1. BFB-Z is an innovative tool that measures in an easy, precise, and fast way the electric balance (energetic homeostasis) that involves the vital capacity, the presence of functional and organic alterations, oxidative stress, and body functions. It is a computerized instrument based on the technique of evoked potentials in easily accessible peripheral electrodermal points placed on the hands and feet. BFB-Z can be considered a variant of the Ryodoraku method, which, as is known, was developed in 1951 Dr. Yoshio Nakatani. The Ryodoraku method is based on the presence of electropermeable points on the body surface. The electrical characteristics of these points, which largely coincide with the main points of classical acupuncture, vary not only with any pathological process but also with the detector probe voltage. Most traditional acupoints may be localized if a 21-volt circuit is used. However, if a 12-volt circuit is used, it is possible to find other electrically conductive points on the body, not associated with specific acupuncture points. Similarly, the BFB-Z analyzes the electropermeability characteristics of 12 skin points (6 from hands and 6 from feet), one for each of the main meridians. Once the electrical characteristics of the 12 points have been measured, the BFB-Z provides a diagram that represents the energetic status of the subject.

### 2.4. Study Design

**Figure 2:**
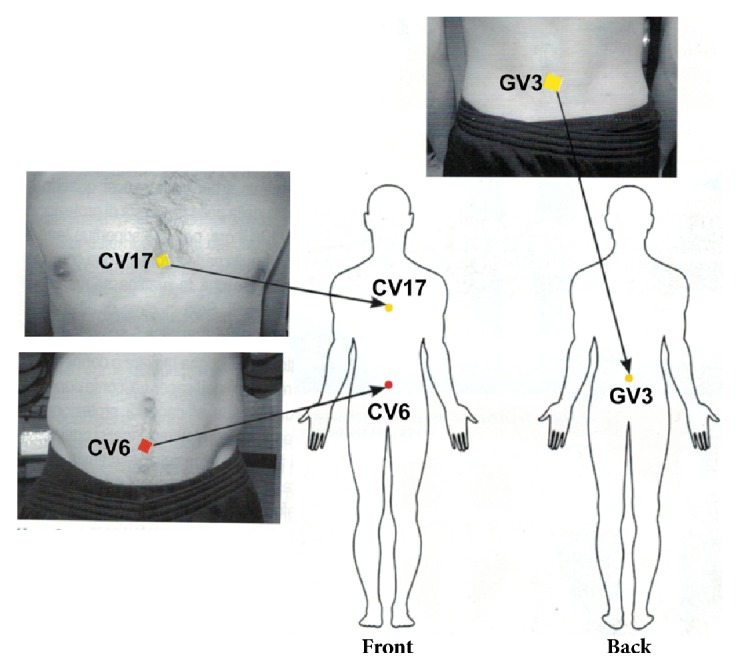
Experimental procedure. A schematic application of Skudo® patches.

All participants were measured at 4 specific time points to evaluate the effectiveness of Skudo® to counteract the electromagnetic field on different level of electromagnetic field using BFB-Z technique. In the first set of experiments 10 participants were measured: at basal level (after 2 days of consecutive work rest) without Scudo®, at basal level (after 2 days of consecutive work rest) with Scudo®, after front positioning to a WiFi antenna which was switch off without Scudo®, after front positioning to a WiFi antenna which was switch on and the electromagnetic waves affect the vertebral column longitudinally with Scudo®, and after front positioning to WiFi transmitter on which the patch is placed. In the second set of experiments, the same 10 participants were measured: at basal level (after 2 days of consecutive work rest) without Scudo®, after at least 10 hours of hospital work in radiology department without Scudo®, and after at least 10 hours of hospital work in radiology department with Scudo®. As illustrated in Figure 2, 3 patches were applied to each participant: 2 at the meridian position CV17 (Conception Vessel 17) and at GV3 (Governing Vessel 3) and 1 at the meridian position CV6 (Conception Vessel 6), as shown in the picture.

### 2.5. Data Processing and Statistical Analysis

The raw data were processed using Prism GraphPad statistical software for normalization, peak picking, and comparison between groups. The images were produced directly by BFB-Z and ImageJ. One-way analysis for variance (ANOVA) with Tukey's post hoc tests was carried out for the comparison between groups, and all results were expressed as mean ± SD. Differences were considered to be statistically significant with p < 0.05.

## 3. Results and Discussion

### 3.1. Analysis of Standard Diagram

**Figure 3:**
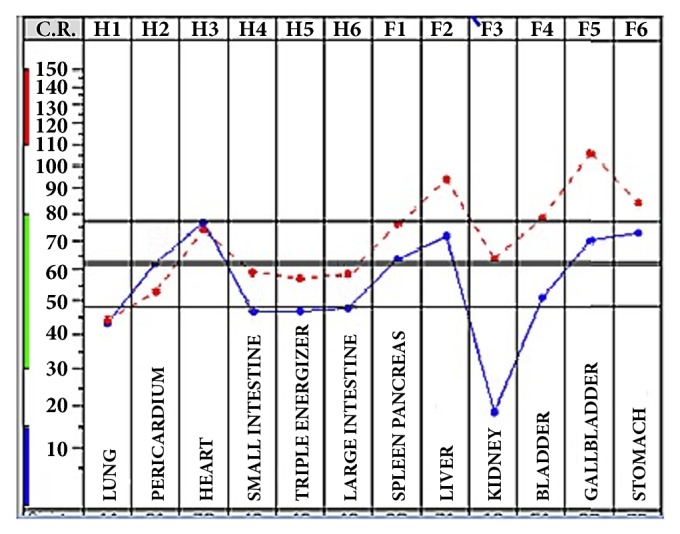
Standard layout obtained by BFB-Z on healthy subject.

The term “biological reactivity” refers to the level of “vital energy of the organism”, which corresponds with Qi of Traditional Chinese Medicine (TCM). An example of standard layout obtained by BFB-Z on healthy subject with good level of bioenergetic reactivity and vegetative balance is shown in Figure 3. In particular, the connection between H and F points of Ryodoraku system and the meridians of Traditional Chinese Medicine should be noted. In the standard diagram taken from a healthy subject, 3 steps of energy increase can be observed: at H3, F2, and F5 points, respectively. In addition, at F3 point, an important subsidence was observed. The meaning of this curve was explained by the correlation with meridians points. Indeed, H3 point corresponds to the fire, the energy of health which was represented by the heart; F2 and F5 points correspond to wood and the energy of blood; finally, F3 point corresponds to the water and the way of expulsion of impure substances and is connected to the metabolism of the body. Following the principles of TCM, the basic elements are five: water, fire, wood, metal, and earth; the relationships between these five elements represent a model of interaction between the internal organs and tissues and sense. The double horizontal line that crosses the whole layout indicates the level of bioenergetic reactivity. Finally, on the left side, there is a green line that corresponds to the normal energy level which is related to age and sex.

**Figure 4:**
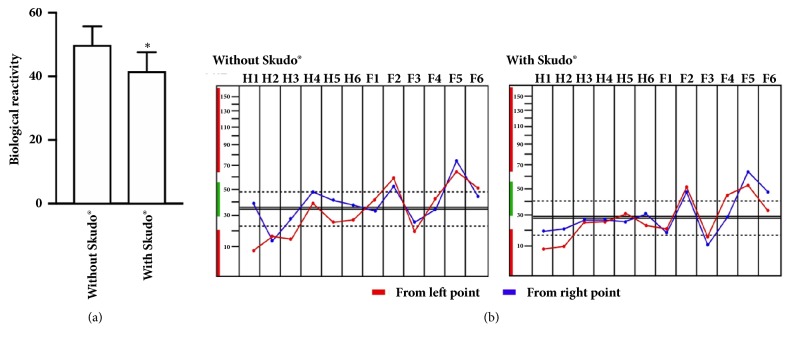
Analysis of the measurements at basal level with or without Skudo®. In (a) the biological reactivity measured by BFB-Z at 10 participants. In (b), an example of layout observed. Data reported are a means+/- SD. *∗* p < 0.05 versus without Skudo®.

For this reason, the experiments were performed starting from analyzing the basal level without and with Skudo® patch. As reported in Figure 4(a), a significant (p < 0.05) reduction in the level of bioenergetic reactivity on all participants was observed in presence of Skudo® patches indicating that the energy of individuals leads to stabilization, to achieve a more stable balance that is closely related to the neurovegetative activity, named as homeostasis. These are important data to support the hypothesis that the neurovegetative activity is a crucial regulator of stress. In addition, in presence of Skudo®, the shape of the layout (Figure 4(b)) demonstrated a better alignment between measurements from left and right points than in absence of Skudo®; these data demonstrated a lower perturbation of the biological impedance of the body and effectiveness of Skudo® into isolate the body from the external environment. Finally, a reduction of stress was also observed, as shown by the plateau phase of H2-H3-H4 points which represent the fire. On the contrary, wood and water elements remain unaffected, indicating absence of interfering effects on metabolism.

### 3.2. Skudo® Protection against EMF

**Figure 5:**
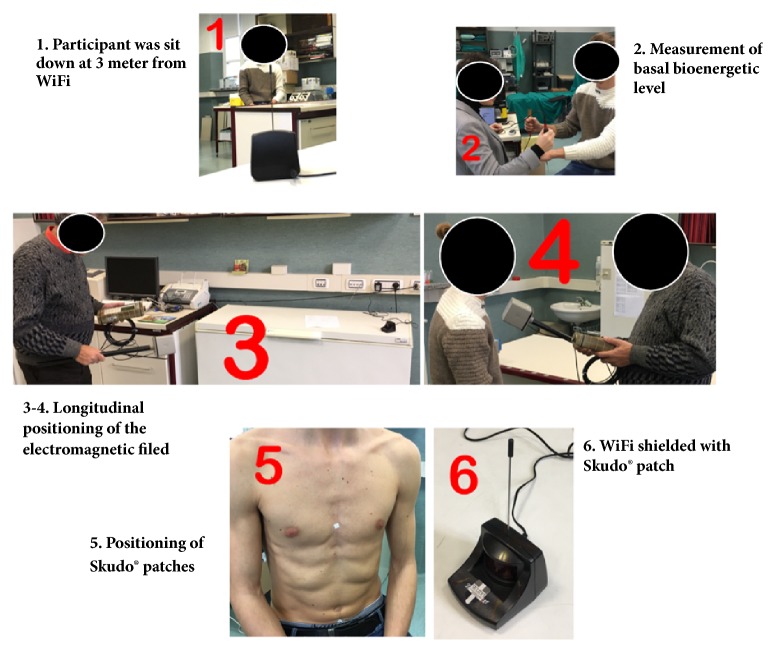
Description of EMF experiments with or without Skudo® patches.

The importance of the barrier from electromagnetic fields was confirmed by the successive experiments performed near a WiFi antenna, as reported in Figure 5 in which each measurements were described.

**Figure 6:**
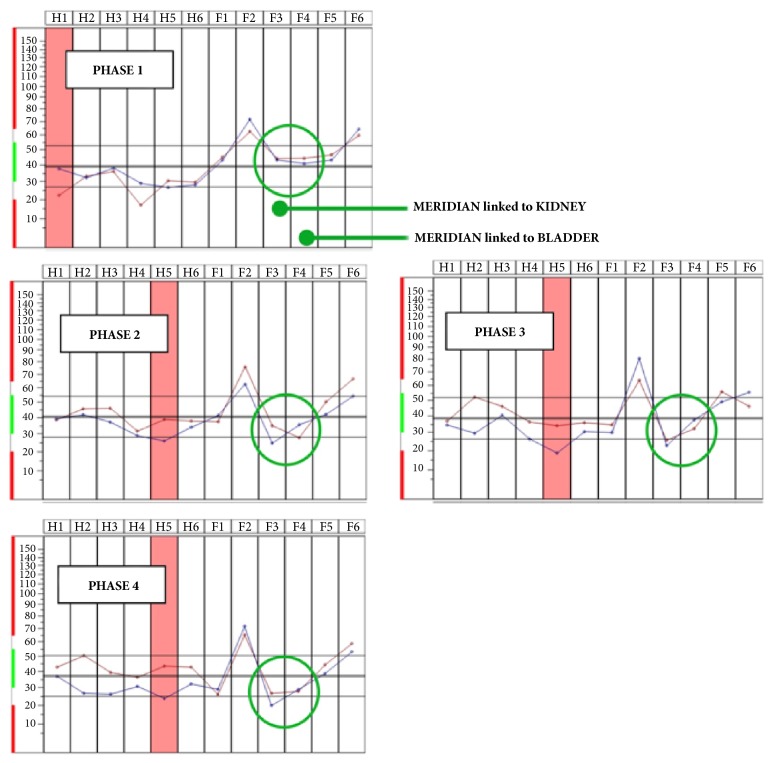
An example of layout obtained during the measurements by BFB-Z of 10 participants.

Analyzing the bioenergetic status of each measurements of participants, an evident unbalance was observed on meridians that regulate water. As reported in Figure 6, two water meridians related to kidney and bladder during the irradiation (phase 2) were unbalanced compared to the basal level (phase 1); the layouts of the left in red and of the right in blue were crossed. This phenomenon could be interpreted as an unbalance of the energy linked to water determined by the electromagnetic field of the Wi-Fi radiation source. Usually in physiological conditions, there are no crossing points of the left and right tracks. This phenomenon is relevant since, placing the Skudo® patches on the body (phase 3) and then on the source of EMF (phase 4), the traces corresponding to left and right side of the body return aligned and therefore are very similar to the basal layout.

### 3.3. Skudo® Protection against SMF

**Figure 7:**
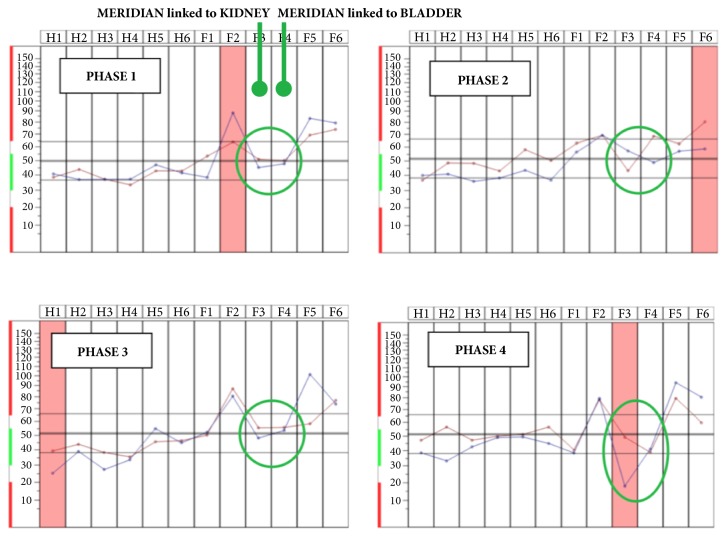
An example of layout obtained during the measurements by BFB-Z of 10 participants.

As has been observed on previous experiments in presence of WiFi antenna, also in these experiments performed during the working in radiological environment and in particular during magnetic resonance, the bioenergetic profiles measured on all participants in the different phases (phase 1, at basal rest; phase 2, working day without Skudo® patches; phase 3, baseline of working day; phase 4, day of work with Skudo® patches) show an imbalance of the meridians responsible for the control of water. As reported in Figure 7, the meridians corresponding to kidney and bladder during phase 2 (irradiation moment without Skudo® patches) have bioenergetic profiles of the left side (showed in red) and of the right side (reported in blue) that cross. This confirms the imbalance of water due to an electromagnetic field. The presence of the patches realigns the traces (phase 4), demonstrating the shielding and balancing capacity of the Skudo patches.

## 4. Discussion

In this study, the protective effects of specific patches on the effects of magnetic fields on the human body were evaluated. The studied devices, commercially available patches called Skudo®, have been placed on some important acupoints. In particular, the selected points were 6CV, 17CV, and 3GV. As known, the three points chosen belong to two of the extraordinary meridians: conception vessel and governor vessel, also known as Ren Mai and Du Mai. The choice of these meridians is motivated by the fact that Ren Mai represents a fundamental level of energetic functioning and has as main action the toning of the Kidney Yin and also nourishes and regulates the Blood. In addition, Ren Mai controls Sea of Yin meridians and circulates Yin Qi, including Blood, Essence, and Body Fluids. For what concerns the Du Mai meridian, its main functions are regulating the circulation of energy and blood in the Yang meridians (hence the name of "Yang meridian sea"), regulating the functional activities of the brain and the marrow spinal, and regulating the function of urinary and reproductive systems. The Governing and Conception Vessels are the main rivers of the body's Yin and Yang energies. They are polar aspects of the body, perfectly complementary, like midnight and midday [21]. The two anterior points chosen for the positioning of the 6CV and 17CV patches correspond to the Lower Dantian and the Middle Dantian, respectively. The Lower Dantian is the major storage area for the various types of Kidney energies. The Kidney energies, in turn, are closely linked with the prenatal energies and provide the foundation for all other types of energy (like Jing, Qi, Yin, and Yang) in the body [21]. The Lower Dantian is connected to the first level of Wei Qi. This level of Protective Qi circulates outside the body, extending roughly two inches beyond the body's tissues. As the Lower Dantian fills with Qi, the Wei Qi field naturally becomes thicker. The Lower Dantian collects Earth energy and is associated with Jing and the energy of the physical body. The Earth energy that is transformed in the Lower Dantian is a dense, full, thick energy. In the above analogy of the transformations of water, the energy in the Lower Dantian relates to ice, the densest state of water. The Lower Dantian acts as a reservoir for heat and energy and is associated with the Kidneys. The Kidneys control the Water element in the body, and, in alchemical terms, Jing is said to be analogous to the water in the cauldron [21]. The third patch is placed on the midline of the lower back, in the depression below the spinous process of the second lumbar vertebra. This region corresponds to one of the most important point: called the Mingmen. It is the centre of vitality and is the point where the original life essence of the individual is based. The* Classic of Difficulties* said* “On the left is the Kidney, on the right is ming men”* whilst according to Zhang Jing-yue* “Ming men resides between the Kidneys”*. The exact location of ming men (Gate of Life) has been described differently at different times, but as its name makes clear, Mingmen is an important area to influence the ming men and the ministerial fire to which it is closely related. As well as influencing the ming men fire, this area located on the Governing vessel has a strong regulatory effect on the yang qi and the exterior portion of the body. For this reason, this area is particularly useful for the treatment of heat disorders, whether interior or exterior, excess or deficient. Treating this area it is possible to drain heat manifesting as ‘heat in the body like fire' [21]. The diagram provided by the BFB-Z by measuring the electropermeability of the 12 sample points shows the bioreactivity status of the two halves of the body, right and left. Furthermore a double horizontal line indicates the level of biological reactivity. As described above, the protection provided by the patches mainly concerns the water (front points) and the focus (back point). For the TCM, water is one of the five fundamental elements of life. As already mentioned, water is managed by the Kidney and the Bladder: the Kidney both as an organ and as a meridian; the Bladder as a bowel and as a meridian. It should be noted that for the TCM the Kidney is not identified with the anatomical organ of modern medicine and its physiology, but with the set of energies expressed by the Water movement. The Kidney of TCM is, among all, perhaps the organ farthest from the kidney of Modern Medicine. Some Western doctors who support an integration between the two medicines have identified the Chinese kidney system with the immune system, endocrine and hormone, with important glands such as the thyroid, parathyroid, and adrenal glands. The Kidneys tesaurize the jing, the ancestral essence, that is the result not only of the union of the masculine with the feminine, but also of the transformation of nourishment and liquids. The jing presides over the development of the organism and represents the vital reserve of energy. The kidneys purify the liquids and reintroduce them into the life cycle. They are the "valves" which, opening and closing, favor the circulation of liquids. As far as the bladder is concerned, its meridian crosses the whole body from head to toe, and, on its back, where the two branches of which it is composed run, its crosses the most important muscular bundles in its path. The bladder is responsible for the distribution of fluids throughout the body, but especially the muscles, but it deals with the elimination of toxins through the liquids. The bladder regulates the active liquids in the body, avoids both dryness and flooding, and guarantees a beneficial humidification to the muscles. The bladder transforms the liquids that come to it; on the one hand it makes their recoverable part rise; on the other it thickens the part with the waste and eliminates it through the urine. Life is based on water that represents its universal support. The human body contains more than 50% by volume, but if we consider the total number of molecules that make up our body, water makes up 99% of the total [22]. Many studies have been carried out on the electromagnetic properties of water and on its behavior when it is exposed to electromagnetic fields [23, 24]. The water molecule, subjected to irradiation, absorbs the energy of the electromagnetic waves, if the latter have a frequency that approaches that of the microwaves, i.e., 2,450 GHz. This absorption results in a vibration of the water molecule that it could interfere with all the metabolic reactions of the cells, from enzymatic activity to protein synthesis, up to the processes of cell replication. Furthermore, a recent study [25] showed that a magnetic field of 1.2 micro-Tesla inhibits the action of melatonin. Another consideration that can be made by analyzing the results of the tests is that the patch on the back protects the body from an imbalance of heat, since the area corresponding to Mingmen. It is well known that magnetic fields, especially variable ones, generate thermal effects that can influence the metabolism [26]. The screen provided by these patches can therefore be effective on two important aspects of biological process control.

## 5. Conclusions

Maintaining a correct Yin/Yang balance through the use of acupoints is undoubtedly a method that allows the energies of the individual to be maintained longer and with a better quality. The true effectiveness of patches application is manifested in its rebalancing and preventive action on the disharmony that leads to the exhaustion of the energies of the individual.

This study shows that both SMF and EMF cause an alteration of the body's water system. The application of the Skudo® patches determines a regularization of the bioenergy levels correlated with the Water System.

The application of Skudo® on the EMF source suppressed the imbalance effect of the Water System found in the subject without any protection.

## Data Availability

All data reported have been obtained from experiments carried out in author's laboratory.
